# Where Next for Microbiome Research?

**DOI:** 10.1371/journal.pbio.1002050

**Published:** 2015-01-20

**Authors:** Matthew K. Waldor, Gene Tyson, Elhanan Borenstein, Howard Ochman, Andrew Moeller, B. Brett Finlay, Heidi H. Kong, Jeffrey I. Gordon, Karen E. Nelson, Karim Dabbagh, Hamilton Smith

**Affiliations:** 1 Division of Infectious Diseases, Brigham and Women’s Hospital, Department of Microbiology and Immunobiology Harvard Medical School and HHMI, Boston, Massachusetts, United States of America; 2 Australian Centre for Ecogenomics and Advanced Water Management Centre, The University of Queensland, Brisbane, Australia; 3 Department of Genome Sciences, University of Washington, Seattle, Washington, United States of America; 4 Department of Computer Science and Engineering, University of Washington, Seattle, Washington, United States of America; 5 Santa Fe Institute, Santa Fe, New Mexico, United States of America; 6 Department of Integrative Biology, University of Texas, Austin, Texas, United States of America; 7 Department of Ecology and Evolutionary Biology, Yale University, New Haven, Connecticut, United States of America; 8 Michael Smith Laboratories, University of British Columbia, Vancouver, Canada; 9 Dermatology Branch, Center for Cancer Research, National Cancer Institute, National Institutes of Health, Bethesda, Maryland, United States of America; 10 Center for Genome Sciences and Systems Biology, Washington University School of Medicine, St. Louis, Missouri, United States of America; 11 J. Craig Venter Institute, Rockville, Maryland, United States of America; 12 Second Genome, Inc., South San Francisco, California, United States of America; 13 J. Craig Venter Institute, La Jolla, California, United States of America

## Abstract

The development of high-throughput sequencing technologies has transformed our capacity to investigate the composition and dynamics of the microbial communities that populate diverse habitats. Over the past decade, these advances have yielded an avalanche of metagenomic data. The current stage of “van Leeuwenhoek”–like cataloguing, as well as functional analyses, will likely accelerate as DNA and RNA sequencing, plus protein and metabolic profiling capacities and computational tools, continue to improve. However, it is time to consider: what’s next for microbiome research? The short pieces included here briefly consider the challenges and opportunities awaiting microbiome research.

## Abstract 2

The last decade has seen a staggering transformation in our knowledge of microbial communities. Here, seven short pieces speculate as to what the next ten years might hold in store.

*This Perspective is part of the “Where next?” Series*.

Soon, we will enter an era when “the number of population genomes deposited in public databases will dwarf those from isolates and single cells” (Gene Tyson). Clearly, as all authors noted in the following, our focus will move from describing the composition of microbial communities to elucidating the principles that govern their assembly, dynamics, and functions. How will such principles be discovered? Elhanan Borenstein proposes that a systems biology–based approach, particularly the development of mathematical and computational models of the interactions between the specific community components, will be critical for understanding the function and dynamics of microbiomes. Evolutionary biologists Howard Ochman and Andrew Moeller want to decipher how microbial assemblies evolve but challenge us to also consider the role of microbial communities in organismal evolution, and they make the exciting prediction that microbes will be implicated in the evolution of eusociality and cooperation. Brett Finlay underscores the need for deciphering the mechanistic bases—particularly the chemical/metabolite signals—for interactions between members of microbial communities and their hosts. He emphasizes how this knowledge will enable creation of new tools to manipulate the microbiota, a key challenge for future investigation. Heidi Kong also encourages deciphering the mechanisms that underlie associations between particular skin surfaces and disorders and their respective microbiota. Jeffrey Gordon considers several intriguing opportunities as well as challenges that manipulation of the gut microbiota presents for improved human nutrition and health. Finally, Karen Nelson, Karim Dabbagh and Hamilton Smith suggest that using synthetic genomes to create novel microbes or even synthetic microbiomes offers a new way to engineer the microbiota. Overall, future microbiome research regarding the molecules and mechanisms mediating interactions between members of microbial communities and their hosts should lead to discovery of exciting new biology and transformative therapeutics.

## A Genome-Centric View of Microbiomes

### Gene Tyson

Over the last decade, metagenomics has had a major impact on the way we study the microbiomes of environmental, clinical, and engineered habitats. Through shotgun sequencing of DNA extracted from microbial communities, metagenomics bypasses traditional culture-dependent biases and holds the promise of genome-based insights into the mostly uncharted microbial world. Until very recently, it has not been possible to obtain near complete genomes from these data because limited sequencing throughput combined with the complexity of the microbial communities under consideration precluded assembly, the only exception being low complexity communities where genome reconstruction of dominant populations has been possible. Therefore, the primary way of extracting biologically meaningful information from largely unassembled metagenomic datasets has been to use gene-centric approaches that explore the distribution and abundance of genes and gene families between different environments. However, such analyses typically only use a tiny fraction of the entire dataset.

Recent advances in high-throughput sequencing and new tools for analyzing metagenomic data are driving the rapid evolution of this field. One of the biggest changes is the increased cost effectiveness and throughput of sequencing, which now makes it feasible to move beyond single snapshot metagenomes to biologically replicated series of related metagenomes [1, 2]. Differential coverage–based approaches for analyzing metagenomic data are resulting in the en masse recovery of tens to hundreds of population genomes from many different environments, including rare populations from high complexity communities. In concert, lineage-specific single-copy marker gene approaches are being developed that allow the completeness and contamination of extracted population genomes to be rapidly assessed. When combined with novel approaches to refine or close population genomes, biological inferences can be made with increased confidence.

Single cell genomics also holds great promise for obtaining genomes from the uncultivated microbial majority [3], although the technically demanding nature of the approach and need for specialized equipment is likely to restrict access to this technology. By contrast, the technology and tools necessary to obtain population genomes from metagenomic datasets is within reach of most laboratories and likely to become widely adopted. It is reasonable to predict, then, that the number of population genomes deposited in public databases will dwarf those from isolates and single cells in the near future. More importantly, these recent developments in metagenomics are leading to a genome- rather than gene-centric view of microbial communities that allows organism-based metabolic network models to be developed. For the first time, we are able to examine the role of individual microbial populations in the context of the rest of the community at the genome, transcriptome, and proteome level. The ability to pull out most of the genomes in any given habitat (with the exception perhaps of soil), combined with the relevant environmental metadata, means that we now have the key information needed to understand and predict ecosystem functionality.

## Systems Biology and Integrative Analysis of the Human Microbiome

### Elhanan Borenstein

The past decade has clearly been a golden age for microbiome research. Extensive efforts to characterize the human microbiome, coupled with exciting advances in sequencing technologies and in computational techniques, have tremendously increased our knowledge about the diversity of the microbiome and about its composition in health and in disease. Arguably, however, much of these recent efforts to map the microbiome and to identify shifts associated with disease are conceptually no different from those described by Antony van Leeuwenhoek, the father of microbiology, over three centuries ago. Indeed, van Leeuwenhoek’s meticulous depiction of the “animals” he observed growing in the scurf of his teeth and his rigorous (albeit in 17th century standards) attempts to compare these animals to those he found on the teeth of women, children, and elderly people, differ greatly in scale from our modern, sequencing-based microbiome surveys but similarly focus on cataloging the components of the microbiome and on comparative analysis. Such detailed surveys are absolutely essential when studying a complex system such as the human microbiome, yet this reductionist approach rarely results in a principled understanding of the system under study. Rather, to truly reveal the underlying principles that govern the human microbiome’s assembly, function, and dynamics ultimately requires going beyond characterizing the composition of the microbiome and calls for the development of systems-level thinking and analysis.

The application of a systems biology approach to microbiome-derived metagenomic data is therefore a natural and critical next step for microbiome research. The development of such a *Metagenomic Systems Biology* framework is already under way, and studies that apply a systems-based analysis to characterize the assembly, organization, and activity of the microbiome have recently been introduced [4, 5]. Following the traditional systems biology paradigm, such studies emphasize the development of mathematical and computational models of the microbiome with a focus on network-based analyses of the interactions between the microbiome’s components (be they genes or species) [6]. Mechanistic and phenomenological models that could provide a predictive understanding of the microbiome’s function and dynamics are an especially promising route, paving the way to rational microbiome design and personalized microbiome-based intervention [7]. Yet, metagenomic systems biology is still in its infancy, and much work is still required to overcome the many challenges involved in modeling a system as multifaceted as the human microbiome and accounting for the complex interplay between the microbiome and its human host.

Importantly, systems thinking, analysis, and modeling are not limited to the study of interactions among various components in the microbiome but should also be applied to studying the links between different facets and measures of the microbiome. Clearly, much of the effort in coming years is likely to focus on generating multiple types of ’omic data to characterize the microbiome, including, most notably, metatranscriptomics, metaproteomics, and metametabolomics [8]. Integrating these meta-omic datasets and, specifically, going beyond the identification of statistical associations and putting forward a systems-level framework that links such data through a comprehensive mechanistic model of the microbiome is probably the biggest challenge facing microbiome research in coming years.

Ultimately, however, just as systems biology research has revolutionized genomics, these efforts to develop an integrative multi-meta-omic systems biology framework and to construct systems-level predictive models of the microbiome are bound to revolutionize human microbiome research and our understanding of this magnificently complex ecosystem.

## Microbiome Evolution

### Howard Ochman and Andrew Moeller

The first high-throughput descriptions of microbial communities provided snapshots of the bacterial diversity occurring at a single place at a single time (and typically recognized with a single gene) but yielded little direct information about what these bacteria were doing, or even if they were alive. We have approached the point where we can make nearly exhaustive surveys of the bacteria inhabiting natural communities; but so far, little is known about their persistence, their patterns of diversification or their episodes of adaptive evolution—basically, why those particular bacteria that are there are there. Within communities, particular taxa rise and fall in abundance, and the causes and consequences of these fluctuations are far from clear. Soon, we will see long-term, in-depth analyses that employ single-cell genomics, instead of simply tallying those lumbering 16S rDNA sequences, to track the evolution and fate of individual bacterial lineages within the complex environment of their hosts. Such studies will reveal how the different evolutionary forces—mutation, selection, migration, and drift—shape the contents, and ultimately, the metabolic capabilities, of microbial communities. Knowledge of these processes will allow us to determine whether the presence and persistence of a bacterial lineage in a microbiome are enabled by the host or by the other microbial members of the community. Furthermore, it will become clear which lineages are functionally relevant to the community and/or the host and which are simply bandwagon microbes.

When considering microbiomes and evolution, there are really two types of questions to ponder. Although we need to understand how evolutionary forces operate in microbial assemblages, the greater mysteries concern those astonishing events through which microbial communities and associations have swayed the course of organismal evolution. Microbes have impelled some of the most profound changes in the evolution of life, and their vast numbers and wide distribution imply the occurrence of several improbable events. Although evolution works in ways that are difficult to predict, we anticipate that microbes will soon be implicated in the evolution of eusociality and cooperation—hailed as one of the major transitions in evolution—having arisen from demands to transfer those much-needed microbes among members of a society.

## Humans and Microbes

### B. Brett Finlay

The past few years have seen a remarkable flurry of findings correlating the microbiota and their impact on many diverse aspects of human biology. However, there are relatively few examples that define the mechanisms by which these interactions occur, and even fewer that then exploit this knowledge. We do not even know yet what defines a healthy microbiota [9]. Most studies of microbiota and disease have relied on community profiling or metagenomics, but these have not been particularly useful in defining mechanisms involved. Two related areas of research in the future will directly address these shortcomings.

The first area, already well underway, is the application of metabolomics to microbiota studies. A wonderful example of this is the demonstration that the short chain fatty acid (SCFA) butyrate, produced by certain Clostridial species, interacts with G protein coupled receptors (GPR41 and GPR43) to modulate regulatory T cell (Treg) production [10]. It is readily apparent how such an approach can then be exploited to improve health or alter disease. However, we know virtually nothing about most microbiota metabolites that are produced in vivo, even though these are the key molecules that microbes use to converse with each other and the host. The ability to grow particular microbes and even microbial communities in culture will enable the identification of such molecules. This knowledge, combined with biological experiments in improved animal models (such as human microbiota transplanted into mice expressing a human immune system) will provide novel tools to study disruption of microbial communication between microbes and between microbes and the host.

The other major area of research that is critically needed is the ability to modulate particular microbes within a microbiota community. Most studies find association with particular microbial species, but this does not prove their involvement. Currently tools to modulate particular microbes are unavailable, although vaccination and phage therapy have been proposed. New methods are needed to tag particular species and/or identify molecules that can impact on specific species. The antibiotic pharmaceutical industry has vast collections of molecules that were originally designed to kill microbes, and repurposing some of these molecules, combined with other creative approaches, may provide such new tools. Complementing this will be new ways to shift microbiota populations. Metabolomic knowledge, as well as the ability to grow defined microbial populations in culture, will certainly provide clues about potential ways to do this. The past few years have seen a remarkable demonstration of the role of microbiota in humans, but what is now needed is a sound understanding of the molecules and mechanisms driving this role and then to capitalize on this knowledge to improve health and decrease disease.

## Varied Biogeography of Skin Microbial Communities in Health and Disease

### Heidi H. Kong

The microbial communities of human skin include signature taxa that are distinguishable from other epithelial sites (oral mucosa, vaginal mucosa, gut), highlighting some of the unique features of the skin microbiome [11]. Recent studies of multiple skin sites in healthy individuals have demonstrated spatial diversity of skin microbial communities: topographically varied microenvironments of oily sites on the head and trunk; moist folds and creases; and dry broad flat surfaces can harbor distinct bacterial communities. In contrast, skin fungal communities are defined primarily by the predominance of the genus *Malassezia* over most of the sampled skin surface, except for the feet, which harbor a more diverse fungal composition [12]. Metagenomics allows investigation into the functional potential of skin microbial communities and demonstrates how species strains can vary biogeographically and individually, likely resulting in functional differences contributing to health and disease [13].

The distinctive features of skin microbiota are also reflected in the compartmentalization of host immune responses to local bacteria, e.g., topical association of commensal *Staphylococcus epidermidis* can elicit IL-17A responses in skin but not in the gut [14]. Many host factors, including physiology, age, metabolism, environment, exposures, and immunity, can have significant effects on the resident skin microbial communities. Further research incorporating many areas of science, e.g. other ’omics approaches, microbiology, molecular biology, and immunology, will be essential to uncover the complexities of skin microbial communities and host–microbial interactions.

An interesting aspect of many skin diseases is the tendency for these disorders to characteristically affect particular skin sites. For example, atopic dermatitis—commonly known as eczema—exhibits site-selective skin eruptions, often affecting certain regions of patients’ arms and legs. In skin microbiome studies of atopic dermatitis patients, the association of *Staphylococcus aureus* and *S. epidermidis* at sites of disease flares raises additional questions about the role of these bacteria in this inflammatory skin condition.

The excitement generated by microbiome studies has fueled tremendous interest in applying knowledge resulting from skin microbiome studies. However, many genomics-based skin microbiome studies to date are associative, and determining the underlying mechanisms responsible for these important observations about skin microbial communities will ultimately be crucial. Premature efforts to manipulate host–microbial homeostasis could create unforeseen adverse outcomes, in light of the potential pathogenicity of many commensal microbes and ability of interspecies interactions to potentiate disease. Further investigations focusing on site-specific skin–microbial interactions will lead to a better understanding of health and skin disorders and subsequently identify potential microbiome-based interventions to ameliorate disease.

## The Nexus of Food, Agriculture, Human Nutrition, and the Gut Microbiome

### Jeffrey I. Gordon

We are witnessing dramatic alterations in how and what we eat. Examples include the move from traditional markets towards branded retail, processed foods with longer shelf lives, more “ready-to-eat” items, the evolving dominance of snacking, and the decline in family dining with the accompanying rise in individual eating. At the same time, we are experiencing rapid expansion of our human population and great challenges related to sustainable agriculture. The coalescence of these forces is creating a need for sustained innovation in identifying affordable new food sources and new highly nutritious foods.

Studies of the human gut microbiome are beginning to have a disruptive effect on current views of human nutrition, and could catalyze efforts to integrate agricultural policies and practice, food production and distribution, and nutritional recommendations for consumers representing different ages, lifestyles, geographies, and states of health. Research platforms are being implemented to determine the effects of existing foods, or those we envision creating in the future, on the gut microbiota and its human hosts [15]. Deeper knowledge of the interrelationship between the foods we consume and the properties of our gut microbial communities should better inform the way we define our nutritional needs and status, thereby emphasizing in new ways how foods are directly linked to human health. This knowledge will provide an impetus to better characterize emerging food consumption patterns in countries representing different cultural traditions, stages of economic development, and land/water resources. It will create an opportunity to differentiate foods based on their effects on different consumer populations with distinct physiological, metabolic, and immune phenotypes, and different gut microbial community configurations. The results may herald a new epoch of precision nutrition, where personal determination of health status (e.g., via smart phone-linked devices or novel single-use diagnostics) drives increased focus on foods as a means for disease prevention as well as treatment.

Agricultural, food, and pharmaceutical companies need to be engaged to effectively and responsibly apply this new knowledge. Government agencies should provide conceptual frameworks for efficient and sensible regulatory schemes, and ways for construing intellectual property that provide appropriate incentives for private investment while protecting the public good.

The ability to improve nutritional status by producing and administering consortia of cultured, naturally occurring members of the gut microbiota of human donors, who may or may not be related to potential treatment recipients by biology or by shared living environments, will focus attention on a number of issues; they include ownership of microbes (and concepts of self) and the type of preclinical data packages and trial designs required for approval of human studies. The possibility of “inoculating” malnourished infants and children with consortia of cultured gut microbes to effect durable repair of their defective gut microbial community maturation and restore healthy growth [16, 17] highlights the need to carefully address issues of short- as well as long-term safety and efficacy and potential societal responses to interventions that produce enduring changes in human biology.

Underlying these efforts is a need to develop an educational outreach with a narrative and vocabulary understandable to a broad and varied consumer population representing different cultural traditions and widely ranging degrees of scientific literacy so that they can make informed choices. It is critical that these scientific, social, cultural, ethical, regulatory, and educational challenges are addressed now, especially in the case of low-income countries where the burden of disease is great and where application of discoveries related to the human gut microbiome are likely to have high impact.

## Synthetic Biology and the Human Microbiome

### Karen E. Nelson, Karim Dabbagh, Hamilton Smith

The field of human microbiome research has exploded since the first publication of a human metagenomic study in 2006 [18]. Current estimates are that the microbes associated with the human body include thousands of strains and species, and contribute significantly more genes than our own (host) genomes. With the advent of next generation sequencing (NGS) and the development of novel bioinformatics approaches, we have gained a deeper understanding of the microbiome and its impact on health and disease in disorders of the skin (psoriasis, acne), the gut (Crohn’s, colon cancer, colitis), the vaginal tract, and the oral cavity (caries). It is likely that research on the human microbiome will continue to demonstrate other correlations such as a relationship with the increased prevalence of autoimmune diseases, including Type 1 Diabetes.

While there have been several promising avenues for development of the microbiome as a diagnostic (PCR-based approaches, for example, for the detection of pathogens), initial approaches to development as a therapeutic are relatively limited. Probiotics containing *Lactobacillus, Bifidobacterium*, and *Saccharomyces* are available in pill form for treatment of traveler’s diarrhea and antibiotic-induced diarrhea. Approaches for other intestinal diseases are much cruder. The most popular example—Fecal Replacement Therapy—relies on the replacement of gastrointestinal microbiota in diseased individuals with the fecal material from a “healthy” individual. Recent studies have suggested that this fecal population can be narrowed to a small number of species, and one could expect that eventually the microbial products that are responsible for alleviating the condition will become a therapeutic product. Microbial replacement therapies will likely become standard practice as we further understand the possibilities, limitations, and implications of the microbiome.

The parallel development and expansion of the field of synthetic genomics creates novel opportunities for synthetic human microbiomes or their synthetic products, which can be used for modulating human health. Synthetic biology has already proved successful in applications to malarial therapeutics and for influenza vaccine production. Synthetic biology approaches create an immense opportunity to develop modified species that can be introduced into the human body and monitored (by use of a water mark or other biomarker). Probiotic or engineered strains can be improved for efficiency—altering pathways that may result in increased production of secondary compounds such as vitamins or the delivery of bioactive payloads. One can imagine yeast, small eukaryotes, or bacteria producing bioactive compounds at disease sites, such as the intestine or on skin, acting as a delivery system. They could also be used to produce synthetic natural-product medicines [19]. Synthetic phages offer the promise of modulating populations of bacteria that have negative effects on human health [20].

While from a commercial standpoint, engineering live microorganisms creates novel intellectual property and products differentiated from existing medicines and therapeutic approaches, one would expect that there will be significant challenges with merging the fields of synthetic biology and human microbiome research from the regulatory perspective, especially with respect to introducing altered species into the healthy human microbiome and natural environment in general. It is a timely topic, warranting further discussion and public awareness as we explore using our natural microbial flora to further benefit our health.

## References

[1] Albertsen M, Hugenholtz P, Skarshewski A, Nielsen KL, Tyson GW, et al (2013) Genome sequences of rare, uncultured bacteria obtained by differential coverage binning of multiple metagenomes. Nat Biotechnol 31: 533–538. 10.1038/nbt.2579 23707974

[2] Imelfort M, Parks D, Woodcroft BJ, Dennis P, Hugenholtz P, et al (2014) GroopM: An automated tool for the recovery of population genomes from related metagenomes. PeerJ PrePrints 2:e409v1 10.7717/peerj.603 25289188 PMC4183954

[3] Rinke C, Schwientek P, Sczyrba A, Ivanova NN, Anderson IJ, et al (2013) Insights into the phylogeny and coding potential of microbial dark matter. Nature 499: 431–437. 10.1038/nature12352 23851394

[4] Greenblum S, Turnbaugh PJ, Borenstein E (2012) Metagenomic systems biology of the human gut microbiome reveals topological shifts associated with obesity and inflammatory bowel disease. Proc Natl Acad Sci U S A 109: 594–599. 10.1073/pnas.1116053109 22184244 PMC3258644

[5] Levy R, Borenstein E (2013) Metabolic modeling of species interaction in the human microbiome elucidates community-level assembly rules. Proc Natl Acad Sci U S A 110: 12804–12809. 10.1073/pnas.1300926110 23858463 PMC3732988

[6] Manor O, Levy R, Borenstein E (2014) Mapping the inner workings of the microbiome: Genomic- and metagenomic-based study of metabolism and metabolic interactions in the human microbiome. Cell Metab. 20: 742–752. 10.1016/j.cmet.2014.07.021 25176148 PMC4252837

[7] Greenblum S, Chiu H, Levy R, Carr R, Borenstein E (2013) Towards a predictive systems- level model of the human microbiome: progress, challenges, and opportunities. Curr Opin Biotechnol 24: 810–820. 10.1016/j.copbio.2013.04.001 23623295 PMC3732493

[8] Segata N, Boernigen D, Tickle TL, Morgan XC, Garrett WS, et al (2013) Computational meta’omics for microbial community studies. Mol Syst Biol 9: 666 10.1038/msb.2013.22 23670539 PMC4039370

[9] Backhed F, Fraser CM, Ringel Y, Sanders ME, Sartor RB, et al (2012) Defining a healthy human gut microbiome: current concepts, future directions, and clinical applications. Cell Host Microbe. 12: 611–622. 10.1016/j.chom.2012.10.012 23159051

[10] Arpaia N, Campbell C, Fan X, Dikiy S, van der Veeken J, et al (2013) Metabolites produced by commensal bacteria promote peripheral regulatory T-cell generation. Nature 504: 451–455. 10.1038/nature12726 24226773 PMC3869884

[11] Huttenhower C, Gevers D, Knight R, Abubucker S, Badger JH, et al (2012) Structure, function and diversity of the healthy human microbiome. Nature 486: 207–214. 10.1038/nature11234 22699609 PMC3564958

[12] Findley K, Oh J, Yang J, Conlan S, Deming C, et al (2013) Topographic diversity of fungal and bacterial communities in human skin. Nature 498: 367–370. 10.1038/nature12171 23698366 PMC3711185

[13] Oh J, Byrd AL, Deming C, Conlan S, NISC Comparative Sequencing Program, Kong HH, Segre JA (2014) Biogeography and individuality shape function in the human skin metagenome. Nature 514: 59–64. 10.1038/nature13786 25279917 PMC4185404

[14] Naik S, Bouladoux N, Wilhelm C, Molloy MJ, Salcedo R, et al (2012) Compartmentalized control of skin immunity by resident commensals. Science 337: 1115–1119. 10.1126/science.1225152 22837383 PMC3513834

[15] Ridaura VK, Faith JJ, Rey FE, Cheng J, Duncan AE, et al (2013) Gut microbiota from twins discordant for obesity modulate metabolism in mice. Science 341: 1241214 10.1126/science.1241214 24009397 PMC3829625

[16] Sela DA, Mills DA (2014) The marriage of nutrigenomics with the microbiome: the case of infant-associated bifidobacteria and milk. Am J Clin Nutr 99: 697S–703S. 10.3945/ajcn.113.071795 24452239 PMC3927697

[17] Subramanian S, Yatsunenko T, Huq S, Haque R, Mahfuz M, et al (2014) Persistent gut microbiota immaturity in malnourished Bangladeshi children. Nature 509: 417–421. 24896187 10.1038/nature13421 PMC4189846

[18] Gill SR, Pop M, Deboy RT, Eckburg PB, Turnbaugh PJ, et al (2006) Metagenomic analysis of the human distal gut microbiome. Science 312: 1355–1359. 10.1126/science.1124234 16741115 PMC3027896

[19] Walker MC, Thuronyi BW, Charkoudian LK, Lowry B, Khosla C, Chang MC (2013) Expanding the fluorine chemistry of living systems using engineered polyketide synthase pathways. Science 341: 1089–1094. 10.1126/science.1242345 24009388 PMC4057101

[20] Citorik RJ, Mimee M, Lu TK (2014) Bacteriophage-based synthetic biology for the study of infectious diseases. Curr Opin Microbiol 19: 59–69. 10.1016/j.mib.2014.05.022 24997401 PMC4125527

